# Characterization of vertigo and hearing loss in patients with Fabry disease

**DOI:** 10.1186/s13023-018-0882-7

**Published:** 2018-08-15

**Authors:** Maria Köping, Wafaa Shehata-Dieler, Dieter Schneider, Mario Cebulla, Daniel Oder, Jonas Müntze, Peter Nordbeck, Christoph Wanner, Rudolf Hagen, Sebastian P. Schraven

**Affiliations:** 1 Department of Oto-Rhino-Laryngology, Plastic, Aesthetic and Reconstructive Head and Neck Surgery, Comprehensive Hearing Center (CHC) and Fabry Center for Interdisciplinary Therapy (FAZIT), University Hospital Würzburg, Josef-Schneider-Straße 11, Haus B2, D-97080 Würzburg, Germany; 2 Department of Internal Medicine I, Comprehensive Heart Failure Center (CHFC) and Fabry Center for Interdisciplinary Therapy (FAZIT), University Hospital Würzburg, Oberdürrbacher Straße 6, D-97080 Würzburg, Germany; 3 Department of Otorhinolaryngology, Head and Neck Surgery “Otto Körner”, University Medical Center Rostock, Doberaner Straße 137-139, D-18057 Rostock, Germany

**Keywords:** Fabry disease, Vertigo, VEMP, Cardiomyopathy, Chronic kidney disease, Lysosomal storage disorder

## Abstract

**Background:**

Fabry Disease (FD) is an X-linked hereditary lysosomal storage disorder which leads to a multisystemic intralysosomal accumulation of globotriaosylceramid (Gb3). Besides prominent renal and cardiac organ involvement, patients commonly complain about vestibulocochlear symptoms like high-frequency hearing loss, tinnitus and vertigo. However, comprehensive data especially on vertigo remain scarce. The aim of this study was to examine the prevalence and characteristics of vertigo and hearing loss in patients with FD, depending on renal and cardiac parameters and get hints about the site and the pattern of the lesions.

**Methods:**

Single-center study with 57 FD patients. Every patient underwent an oto-rhino-laryngological examination as well as videonystagmography and vestibular evoked myogenic potentials (VEMPs) and audiological measurements using pure tone audiometry and auditory brainstem response audiometry (ABR). Renal function was measured by eGFR, cardiac impairment was graduated by NYHA class.

**Results:**

More than one out of three patients (35.1%) complained about hearing loss, 54.4% about vertigo and 28.1% about both symptom. In 74% a sensorineural hearing loss of at least 25 dB was found, ABR could exclude any retrocochlear lesion. Caloric testing showed abnormal values in 71.9%, VEMPs were pathological in 68%. A correlation between the side or the shape of hearing loss and pathological vestibular testing could not be revealed.

**Conclusions:**

Hearing loss and vertigo show a high prevalence in FD. While hearing loss seems due to a cochlear lesion, peripheral vestibular as well as central nervous pathologies cause vertigo. Thus, both the site of lesion and the pathophysiological patterns seem to differ.

**Electronic supplementary material:**

The online version of this article (10.1186/s13023-018-0882-7) contains supplementary material, which is available to authorized users.

## Background

Fabry disease (FD) is an X-linked lysosomal storage disorder which is characterized by a reduced or absent enzyme activity of α-galactosidase A. This leads to an intralysosomal accumulation of globotriaosylceramid (Gb3), which results in tissue damage of kidneys, heart and the nervous system [1–4]. The incidence of FD was previously stated at 1:40.000 to 1:117.000 [5, 6], whereas recent studies assumed a much higher occurrence with demographic and ethnical correlation as newborn screenings in Taiwan or Italy suggest [7–9]. Hemizygous males are usually affected more seriously than heterozygous women [10, 11]. The accumulation of Gb3 in kidneys, heart and the nervous system lead to progressive kidney failure, cardiomyopathy and Fabry-associated pain or stroke [12–14]. Consequently, life expectancy is reduced by 15–20 years due to end-stage complications as sudden cardiac death or renal failure [10, 11]. Another, yet poorly understood, organ involvement is of the cochleovestibular system leading to progressive asymmetric hearing loss, tinnitus and vertigo [15–17]. Histological temporal bone findings showed hyperplastic mucosa and seropurulent effusion in the middle ear, strial and spiral ligament atrophy and loss of outer hair cells. A Gb3-storage in spiral ganglia could not be found. There were no pathological findings in sacculus, utriculus or semicircular canals [18].

Since the introduction of enzyme replacement therapy (ERT) in 2001, a reduction of Gb3 storage in kidneys and the heart could be shown [19–22]. Furthermore, clinical data suggest a beneficial effect of ERT in stabilizing hearing loss and improving vestibular function [16, 17, 23].

Despite the profound impact of hearing loss and vertigo on patients’ individually experienced health-related quality of life, comprehensive data supporting the development of new guidelines for the monitoring and treatment of Fabry disease remain scarce due to prognostic domination of other organs. The aim of this current study was to evaluate the prevalence and characterize the patterns of vertigo and hearing loss in dependence of other Fabry-typical organ manifestations and to get hints about the site and the pattern of the lesions.

## Methods

### Subjects

Fifty-seven FD patients (27 male, 30 female; 46,2 +/− 13,8 years, range 19–77 years), who attended the Department of Oto-Rhino-Laryngology, Plastic, Aesthetic and Reconstructive Head and Neck Surgery in Würzburg, were investigated between 04/2012 and 11/2016. Informed oral and written consent had been obtained appropriate to the decision of the institutional review board of the medical department Würzburg (20,170,904 01; 220/15_z). All patients were recruited from the Würzburg Fabry Center for Interdisciplinary Therapy (FAZIT) within the scope of routine check-ups irrespective of any ENT symptoms or comorbidities. Inclusion criteria were age ≥ 18 years and confirmed diagnosis of FD by DNA testing and α-galactosidase A assay.

### Clinical examination

Medical history was taken of all patients before a complete oto-rhino-laryngological examination. Especially, they were asked about hearing loss, tinnitus and vertigo as well as ototoxic medication, noise exposure or infections.

Glomerular filtration rate (CKD-EPI equation) was measured to estimate renal function with following graduation: ≥90, 60–89, 30–59 and ≤ 29 ml/min/1.73 m^2^ [24]. Cardiac function was classified by NYHA score (class 1: no limitation of physical activity; class 2: slight limitation, ordinary physical activity results in fatigue, palpitation or dyspnea; class 3: marked limitation, comfortable at rest, less than ordinary activity causes fatigue, palpitation or dyspnea; class 4: unable to carry out any physical activity without discomfort, symptoms of heart failure at rest) [25]. Patients with no structural cardiac disease were distributed to class 0. Serum lyso-Gb3 (reference: < 0.9 ng/mL) were measured by Centogene (Rostock, Germany) as potential indicator for disease severity [26].

### Audiological measurements

Audiological measurements were performed with calibrated instruments in a sound-proofed room (DIN EN ISO 8253). The audiological evaluation included standard pure-tone audiometry (air conduction AC: 0.25 through 8 kHz; bone conduction BC: 0.5 through 6 kHz), conducted with a clinical audiometer in 5-dB steps. Hearing thresholds were then averaged in 4-pure tone average (4-PTA: 0.5, 1, 2, 4 kHz) and a modified 6-pure tone average (6-PTA: 0.5, 1, 2, 4, 6, 8 kHz), summarizing all values and dividing by 4 resp. 6, so every threshold carries equal weight. Values 10 dB above normative hearing thresholds were considered abnormal (calculation based on [27]).

Otoacoustic emissions (Etymotic ER10, Illinois, USA) were performed in each patient. Furthermore, auditory brainstem response audiometry (ABR) was performed using Eclipse - ASSR EP15/EP25 (Interacoustics, Middelfart, Denmark) in 56/57 patients. Click stimuli were presented at intensities between 10 and 100 dB HL and responses were then averaged and the ABR threshold was visually determined where wave V showed the smallest response amplitude.

### Vestibular measurements

Videonystagmography (VNG) with recording of spontaneous nystagmus (SPN) and caloric testing with warm (44 °C) and cold (30 °C) water or air (Videonystagmograph VNG ULMER, Synapsys SA, Marseille, France) were performed. Results were considered abnormal when canal paresis factor (CP) was above 25%. Cervical Vestibular Evoked Myogenic Potentials (cVEMPs) were recorded ipsilaterally from the tonically activated sternocleidomastoid muscle by surface electrodes. Ocular VEMPs (oVEMPs) were detected contralaterally by surface electrodes inferior the eye, while the patient was looking upward. Stimulation was carried out with monaural clicks of 100 dB and a rate of 5,1 Hz each via insert tips. Each measurement was performed twice and results averaged.

### Statistical significance

A normal distribution was not found using Shapiro-Wilk test, so Kruskal-Wallis test and pairwise Wilcoxon’s rank sum test were applied. Statistical significance was set at the 95% confidence level and above (*p* < 0.05).

## Results

All 57 patients had normal otoscopy results. Two patients were wearing hearing protection at work because of noise exposure, 1 man had history of an acute acoustic trauma. Other risks concerning inner ear damage could not be revealed. Hearing loss has been reported by 35.1% of all patients (unilateral: 4 men / 6 women; bilateral: 10 men) at the time of examination. Of these 20 patients, 18 (90%) complained about one or more episodes of sudden hearing loss which was asymmetric, and 2 patients reported slowly progressive hearing loss. Another 5 patients described hearing loss in the past, which had subjectively fully recovered. Tinnitus was described by 43.9% (male 15, female 10). Vertigo was reported by 54.4%, whereof 17 patients had intermittent, 2 had permanent and 12 had vertigo which could be triggered. In 15.8% vertigo was described as rotary and in each 19.3% it was named staggering or non-directional (Table 1). Seventeen persons did not have any of the symptoms named above.

**Table 1:** Characteristics in history of vertigo (*n* = 57)

	Male	Female	Total (%)
Patients (*n*)	27	30	57 (100)
Vertigo			
Yes	16	15	31 (54.4)
No	11	15	26 (45.6)
Duration			
Intermittent	8	9	17 (29.8)
Permanent	1	1	2 (3.5)
Triggered	7	5	12 (21.1)
Character			
Rotary	4	5	9 (15.8)
Staggering	6	5	11 (19.3)
Nondirectional	6	5	11 (19.3)

At the date of examination 35 patients received ERT (male 21, female 14) with a mean period of time on medication of 5.97 years (range 1–15 years).

Renal function was determined with the eGFR grouped according to KDIGO categories. Twenty-one subjects showed a value of ≥90, 20 patients a value of 60–89, 13 patients a value between 30 and 59 and 3 male patients a value of ≤29 ml/min/1.73 m^2^. According to the NYHA score, 22 subjects were scored within class 0, 13 patients in class 1, 16 patients in class 2 and 6 patients in class 3. 38 patients were assigned to the ‘classical’ FD mutation group and 7 patients had a ‘late onset’ mutation like N215S; 12 patients had mutations that – based on current knowledge – could not be specified.

Pure-tone audiometry showed an asymmetric high-frequency sensorineural hearing loss. Conductive or combined hearing loss did not occur. In 42 patients (73.7%) we found a sensorineural hearing loss ≥25 dB HL in at least one frequency while only 18 (42.9%) of these patients subjectively had symptoms of hearing loss. Men were affected more severely than women. In two patients who reported hearing disability we found normal values in pure-tone audiometry < 25 dB HL in all frequencies.

4-PTA of the better ear according to the WHO classification of disability due to hearing loss showed pathological results (> 25 dB) in 6 patients (10.5%, mean 13.7 dB). Inspecting the bad ear by reason of a markedly asymmetric hearing loss, 17 patients (29.8%) showed at least a slight impairment (mean 22.5 dB, SD 20.1). In higher frequencies above 2 kHz, the degree of hearing loss was significantly depending on the severity of renal and cardiac function (measured by GFR, NYHA, see also Additional file 1). These observations were even more pronounced regarding 6-PTA better reflecting higher frequencies (mean 29.1 dB, SD 22.0).

Comparing 4-PTA thresholds to age specific median thresholds of healthy persons, FD patients show highly increased thresholds after adjusting for age.

Serum lyso-Gb3 levels had no influence on 4-PTA values (*p* = 0.0863 and r^2^ = 0.00053). The categorization of the patients to the groups ‘classical’ versus ‘late onset’ mutations also did not reveal any difference.

Click-ABR analysis revealed normal interpeak latencies I-III, III-V and I-V in all patients, so retrocochlear lesions could be excluded. According to progredient renal and cardiac dysfunction (GFR, NYHA), a statistically significant increase of ABR-thresholds could be demonstrated between subgroups (see also Additional file 2).

**Fig. 1:**
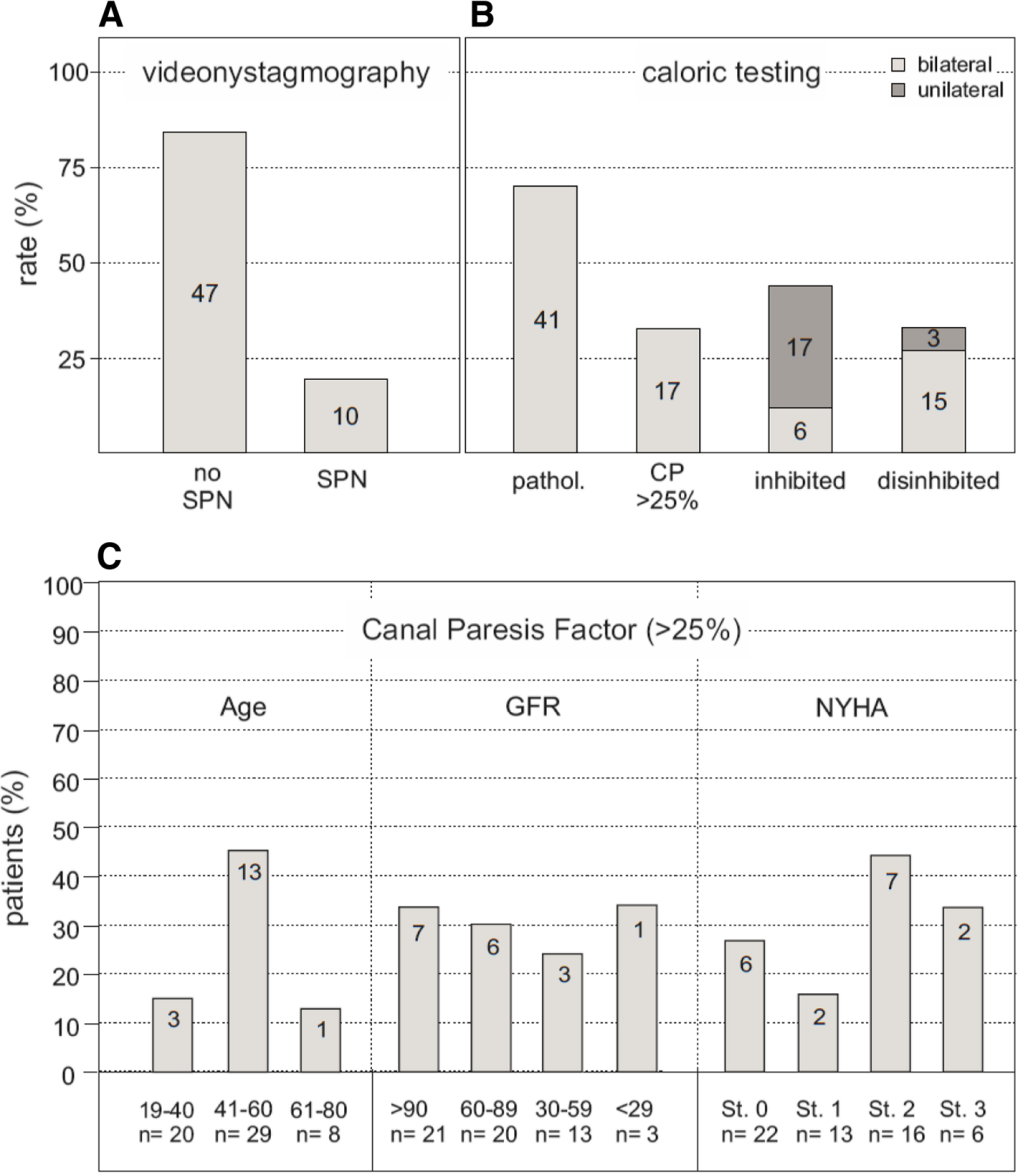
VNG (**a**) 10 out of 57 patients (17.5%) showed a SPN in VNG. (**b**) VNG was pathological in 41 cases: 17 times CP > 25%, 23 times vestibular inhibition and 18 times inhibitory deficit. (*n* = 57). (**c**) CP compared with age groups (19–40,41–60 and 61–80 years), with GFR (divided into groups: ≥90,60-89,30–59 and ≤ 29 ml/min/1.73m^2^) and with degree of heart failure (divided into NYHA classes: 0,1,2,3). (*n* = 17)

VNG (Fig. 1a/b) only was inconspicuous in 16 patients showing normal and equilateral caloric reaction. In 10 FD patients (17.5%) an SPN was detected and in 41 patients (71.9%) a pathological nystagmus was found (limit values ≥1.2 Hz and ≤ 2.1 Hz) after caloric stimulation. An inhibited vestibular function was detected in 40.4% (17 x unilateral with CP > 25%, 6 x bilateral with the sum of all velocities of slow phase < 20°/s). In contrast, 31.6% showed a central disinhibition with frequencies ≥1.2 Hz (3 x unilateral, 15 x bilateral). Age was not associated with an increase of pathological CP. For example, in the subgroup ‘41–60 years’ 13 out of 29 patients (44.8%) had a pathological CP above 25% whereas in subgroup ‘61–80 years’ there was just one out of 8 (12.5%). Different from results in audiological measurements, there could not be found a significant correlation neither with increasing cardiac nor renal impairment (Fig. 1c).

VEMPs were measured in 50 FD patients (24 male, 26 female). Of these, 26 reported vertigo and 24 were free of symptoms. CVEMPs weren’t derivable in 5 ears. Mean latencies of p1/p13 were at 12.0 ms and of n1/n23 at 21.2 ms. Peak-to-peak amplitudes (n1-p1) were 45.5 μV. Pathological cVEMPs values were found in 24 individuals, which can indicate a malfunction of sacculus respectively the inferior vestibular neve: a reduction of amplitude was seen in 17 patients, an extension of latency in 12 patients (partly with overlaps).

OVEMPs could not be elicited in 10 patients (20%), which was not rated as pathological as in previous studies a lack of oVEMPs in up to 50% of healthy individuals was already described [28]. Unilaterally, oVEMPs were underivable in 5 ears. Mean latencies of n1/n10 were 12.4 ms and of p1/p15 17.5 ms. Amplitudes (n1-p1) were at 1.8 μV. Pathological findings occurred in 22 patients, indicating a lesion in utriculus or superior vestibular nerve: a reduction of amplitude presented in 12 patients, an extension of latencies in 13 people (partly with overlaps).

With increasing age (divided in groups 19–40, 41–60 und 61–80 years), a prolongation of latencies and decrease of amplitudes in cVEMPs and oVEMPs were detected (Table 2).

**Table 2:** Latencies and amplitudes with increasing age

Age (years)	cVEMP			oVEMP		
	Latency P1 (ms)	Latency N1 (ms)	Amplitude (μV)	Latency P1 (ms)	Latency N1 (ms)	Amplitude (μV)
19–40	11.73	21.73	58.8	16.44	11.51	2.04
SD	1.66	2.49	35.51	3.64	2.62	1.33
41–60	12.02	21.21	42.36	17.72	12.63	1.62
SD	2.89	3.54	22.94	3.52	2.89	1.23
61–80	12.61	20.3	23.77	18.39	13.57	1.38
SD	2.44	2.87	9.79	3.76	3.84	0.86

**Fig. 2:**
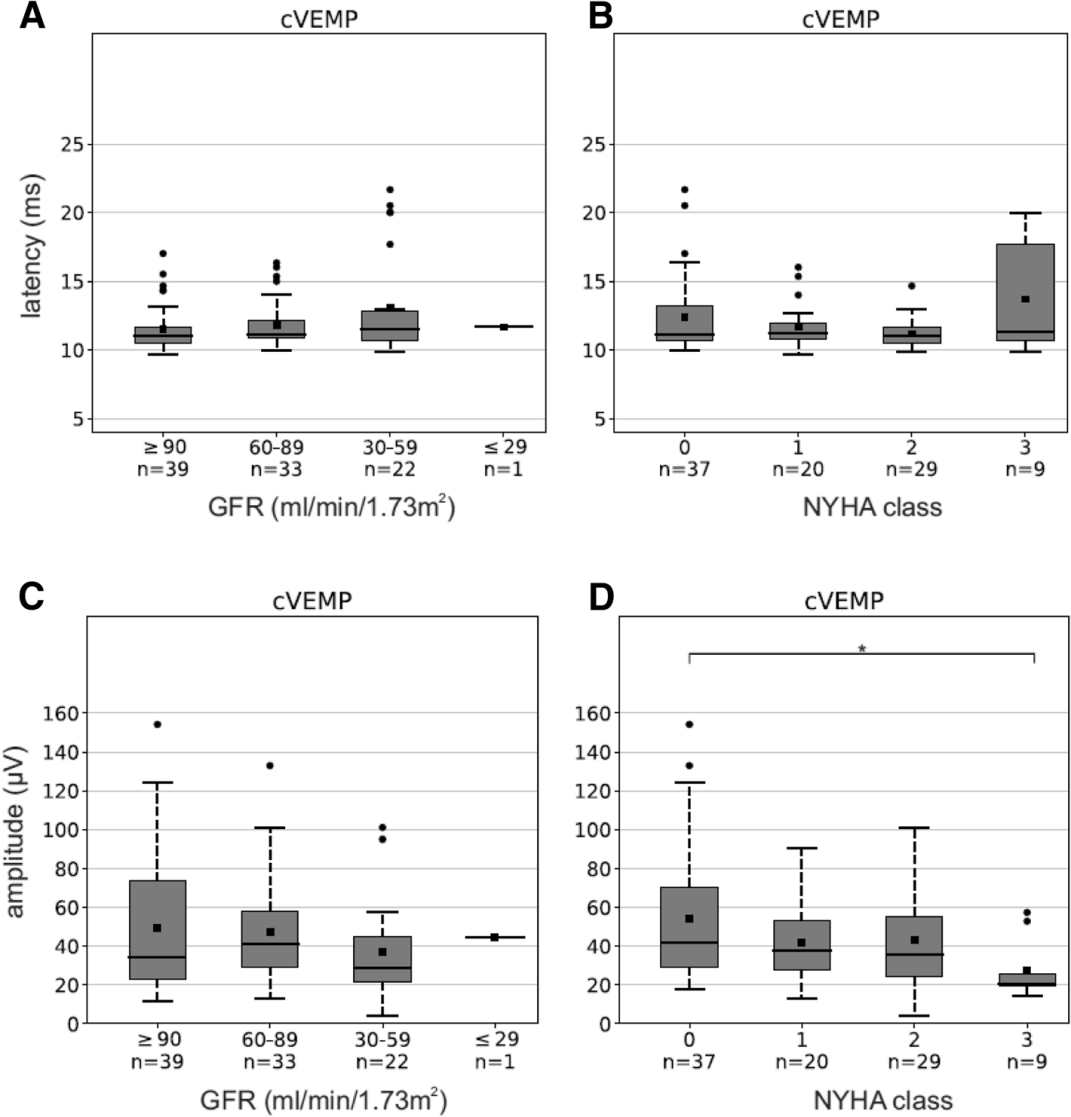
cVEMP latencies and amplitudes vs. GFR and NYHA. (**a**) A decrease in GFR (≥90,60-89,30–59 and ≤ 29 ml/min/1.73m^2^) and (**b**) an increase in NYHA class (0, 1, 2 and 3) tendentially show a prolongation of p1 latencies. (**c**) A decrease in GFR and (**d**) an increase in NYHA are only partially correlated significantly with a decrease in cVEMP amplitude. Asterisks mark significant values with *p* < 0.05. (*n* = 95)

Depending on the severity of renal failure (measured by GFR) respectively cardiac insufficiency (rated on NYHA class), a partly significant decrease of cVEMP and oVEMP amplitudes (peak-to-peak n1-p1) was found. A prolongation of p1 latency (cVEMPs) and n1 latency (oVEMPs) could tendentially be depicted, not being significant (Fig. 2a**-**d showing only cVEMPs).

Lyso-Gb3 levels also had no influence on cVEMP and oVEMP values (e.g. cVEMP p1 latency *p* = 0.91 and r^2^ = 0.0121; oVEMP n1 latency *p* = 0.93 and r^2^ = 0.0006). There likewise was no difference seen between patients with ‘classical’ and ‘late onset’ mutations.

**Fig. 3:**
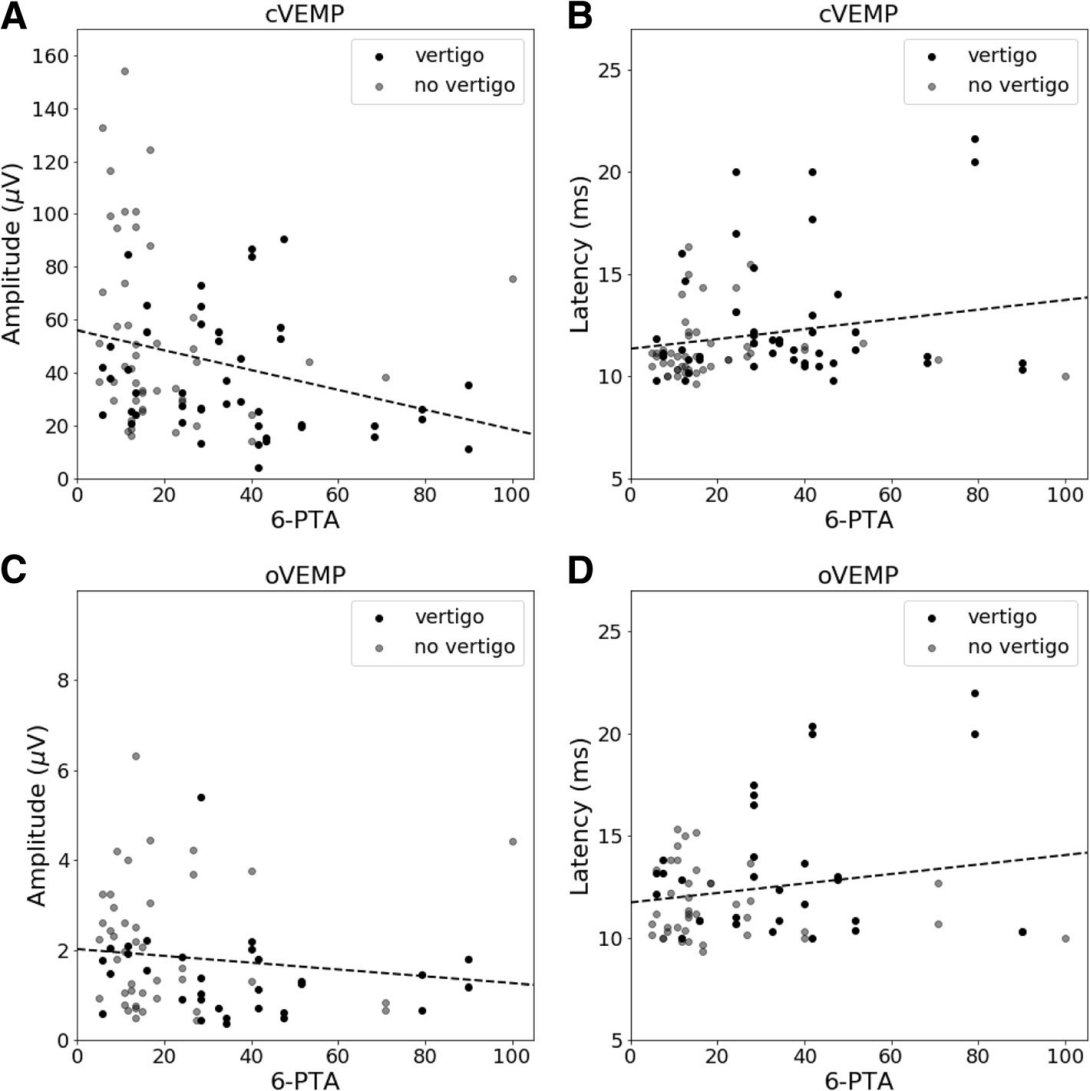
VEMPs vs. 6-PTA. The 6-PTA correlates significantly with the cVEMP amplitude (**a**). The correlation of the 6-PTA with cVEMP-p1 latency (**b**), oVEMP amplitude (**c**) and oVEMP-n1 latency (**d**) is not significant

Modified 6-PTA as parameter of hearing loss revealed a significant negative correlation with the amplitude of cVEMPs in linear regression analysis (Fig. 3a). A lower hearing level (i.e. higher 6-PTA) thus correlated significantly with the reduction of cVEMP amplitude (*p* = 0.011; r^2^ = 0.069). There was also an increase in latencies (Fig. 3b), which was not significant (*p* = 0.051; r^2^ = 0.041). Regarding oVEMPs, a decrease in amplitude and an increase in latencies (Fig. 3c/d) was also observed with increasing 6-PTA. However, the correlation wasn’t significant (*p* = 0.261; r^2^ = 0.018 or *p* = 0.103; r^2^ = 0.036). The results suggest that patients who suffer from sensorineural hearing loss are more likely to have vestibular lesions. In patients with vertigo, smaller amplitudes (cVEMPs: vertigo: mean 37.66 μV, SD 22.07; no vertigo: mean 53.84 μV, SD 34.12; oVEMPs: vertigo: mean 1.41 μV, SD 0.93; no vertigo: mean 2.12 μV, SD 1.39) as well as higher latency values (cVEMPs: vertigo: mean 12.59 ms, SD 3.02; no vertigo: mean 11.44 ms, SD 1,53; oVEMPs: vertigo: mean 13.40 ms, SD 3.39; no vertigo: mean 11.58 ms, SD 1.66) were registered than in those without vertigo.

## Discussion

In this group of 57 FD patients, a high incidence of sensorineural high-frequency hearing loss (73.7%) was apparent. Previously published studies, mostly small case studies, reported an incidence ranging between 19 and 87% [11, 17, 23, 29], but also depicting the high-frequency character and confirming that men are affected more severely than women. In this cohort, patients regularly described that hearing loss had occurred in one or several sudden episodes. In the literature, there are controversial data regarding the incidence of sudden hearing loss. Compared to the results of Ries et al. [30] who reported 10% experiencing hearing loss in the context of an acute event we had a much higher rate (90%). However, other publications support our findings: Conti et al. [16] reported a sudden onset or progression of hearing loss in 6 of 7 patients, and Germain et al. [31] found 7 patients with sudden hearing loss out of 12 patients in total with hearing loss.

The severity of hearing loss is significantly correlated to the function and injury of both kidney and the heart [32]. One limitation might be seen in age-dependency of GFR and NYHA class. Thus, future studies are mandatory to evaluate age- and gender-related control groups for GFR resp. NYHA as well as genotype-specific severity of hearing loss and vertigo. After adjusting for age, FD patients showed markedly increased 4-PTA thresholds in comparison to healthy controls. A confounding presbyacusis could be ruled out. This resembles findings from previous studies [32].

Since retrocochlear pathology could be excluded by ABR, it is supposed that the lesion is located in the inner ear [15, 16]. This is confirmed by the histological results of Schachern et al., who described morphologically regular ganglion cells, which were reduced in the basal turn of the cochlea, as well as an atrophic spiral ligament and stria vascularis [18]. Apart from that, vascular damage by lysosomal Gb3-storage in endothelial cells or by smooth muscle cell proliferation with consecutive infarction of small vessels, is a possible reason [1].

Vertigo seems to follow a more complex pattern. The incidence of vertigo was 54.4% and could occur separately or in combination with hearing loss or tinnitus. FD patients showed a higher incidence compared to a telephone survey with more than 8000 participants conducted in Germany in 2003, where 22.4% of men and 36.2% of women reported having suffered at least once from dizziness [33]. Data about the presence of vertigo in FD vary between 25 and 33% [34, 35].

In 71.9% pathological nystagmus reactions were recorded in VNG and CP was increased > 25% in 29.8%. Most common findings were a one-sided reduction, which suggests a peripheral vestibular lesion, or a bilateral inhibitory deficit, which is more an expression of a central genesis. Other authors showed abnormal VNG results between 17.5% [36] and 21% [17]. In direct comparison it appears that we detected substantially more pathological values. One has to note, however, that we have focused not only on a pathological CP but also on bilateral pathologies like bilateral inhibition and disinhibition.

CVEMPs were pathological in 48% and oVEMPs in 44%. Amplitude reduction as sign of peripheral vestibular or otolithic origin was observed 17 times in cVEMPs and 12 times in oVEMPs. An extension of latency as sign of a neurogenic or central pathology was found 12 times in cVEMPs and 13 times in oVEMPs. These results are comparable to a recently published study with a mixed-gender group of 36 Fabry patients [37]. For the first time, it was reported on pathological cVEMPs in 45% of the patients, also occurring in nonsymptomatic patients. VNG showed abnormalities in 51% and audiometry in 58% of the cases.

Increasing renal or cardiac dysfunction did not lead to significant changes in VNG. In VEMPs, a tendency towards amplitude reduction or latency extension was observed, although only partially significant. However, individual groups (GFR / NYHA) in some cases had very different distributions. In future work, further data collection for more representative groups is important.

Although serum lyso-Gb3 has been revealed to be an important biomarker for staging FD [26], lyso-Gb3 levels did not correlate with cochlear or vestibular affection. Furthermore, it might be speculated whether central lesions such as stroke are related to neurootological symptoms. An association had already been suggested in previous studies [30]. In this study, there were only 4 patients with stroke in the medical history. Even though 50% of these had pathological audiological findings and all had vestibular deficits, a valid statistical analysis was not possible due to the small case number. Additional collection of data and the correlation with stroke and asymptomatic MRI lesions therefore will be of major interest in future studies.

Several studies have shown that the type of mutation substantially affects organ involvement [1, 38, 39]. Nevertheless, in the current patient collective we could not prove a correlation with the severity of audiological or vestibular damage. Whether specific mutations might however still account for clinically relevant effects on the vestibulocochlear function is the subject of further research.

Of course, also other common and non Fabry-related reasons of dizziness like cardiac insufficiency [40] need to be taken in account and excluded. Moreover, it should be noted that the applied neurootological tests did mainly investigate the lateral semicircular canal as well as sacculus and utriculus. The testing of posterior and anterior semicircular canal using video head impulse testing could further enhance the diagnostic setting and are planned for further evaluations.

VEMPs should always be interpreted in the overall context and, if possible, combined with other vestibular tests like VNG or video head impulse testing. Since values in individuals vary relatively widely and depend on the type of stimulus (click / burst, AC / BC) and the intensity of stimulation, narrowly set standard values are difficult to ascertain [41–44]. However, intraindividually, values are quite constant [45], so that VEMPs are suitable for the identification of side differences and for monitoring the progression. Age-related influences also play a role: with increasing age, amplitudes of cVEMPs and oVEMPs decrease and latencies increase slightly [42]. This could be confirmed in this work.

At the time of presentation, 35.1% of all FD patients perceived subjective hearing loss, 56.9% reported dizziness symptoms and 28.1% reported a combination of both symptoms. However, it was remarkable that a simultaneous onset of symptoms could rarely be indicated. In addition, 33.3% of all patients noticed hearing impairment in the sense of an acute hearing loss, whereas only 2.9% reported a sudden onset of dizziness, as in the case of a vestibular neuritis. On the other hand, in cases of common presence, there was no correlation between the side of a measurable hearing loss and the side of a pathological caloric or electromyographic measurement. This raises the question whether these are different types of lesions or different pathophysiological causes (cochlear, vestibular, vascular, neurogenic). Other authors reached similar results [17, 36], not finding a connection between audiological and vestibular symptoms and assuming different pathophysiological patterns. Similarly, Conti and Sergi [16] showed unilateral cochlear and bilateral vestibular abnormalities in a group of 14 mixed-gender FD patients, which did not occur more frequently in combination.

In contrast to the audiological results, a clear pathophysiological pattern could not be identified in the diagnosis of vertigo, so that combined peripheral and central vestibular pathologies have to be assumed. Further clinical and pathohistological studies are necessary to decipher the pathophysiology of vestibulocochlear symptoms in Fabry’s disease.

## Conclusion

High-frequency hearing loss and vertigo are common in FD patients. Hearing loss is due to a cochlear lesion without any signs of retrocochlear pathology. Vertigo seems to be caused by peripheral vestibular as well as central nervous pathologies. The site of lesion and the pathophysiological pattern seem to differ. Every FD patient should obtain an extensive audiological and vestibular testing regularly.

## Additional files

Additional file 1: Pure Tone Audiometry in relation to renal and cardiac function. Pure Tone Audiometry (AC) of the bad ear shows that hearing loss above 2 kHz is depending on the grade of GFR and NYHA. (PDF 1355 kb)

Additional file 2: ABR thresholds vs. GFR and NYHA. According (A) to progredient renal dysfunction (GFR, divided into groups: ≥90,60-89,30–59 and ≤ 29 ml/min/1.73m2) and (B) cardiac dysfunction (NYHA, divided into classes: 0,1,2,3), a statistically significant increase of ABR thresholds could be demonstrated between single groups. (PDF 1390 kb)

## Abbreviations

ABR: Auditory brainstem response
AC: Air conduction
BC: Bone conduction
CP: Canal Paresis Factor
ERT: Enzyme replacement therapy
FAZIT: Würzburg Fabry Center for Interdisciplinary Therapy
FD: Fabry disease
Gb3: Globotriaosylceramid
GFR: Glomerular filtration rate
NYHA: New York Heart Association
PTA: Pure tone average
SPN: Spontaneous nystagmus
VEMPs: Vestibular Evoked Myogenic Potentials
VNG: Videonystagmography
