# Supplementary figures and images for: Characterization of vertigo and hearing loss in patients with Fabry disease
Source: Orphanet J Rare Dis. 2018 Aug 15;13:137. doi: 10.1186/s13023-018-0882-7 (PMC6094894; doi:10.1186/s13023-018-0882-7)

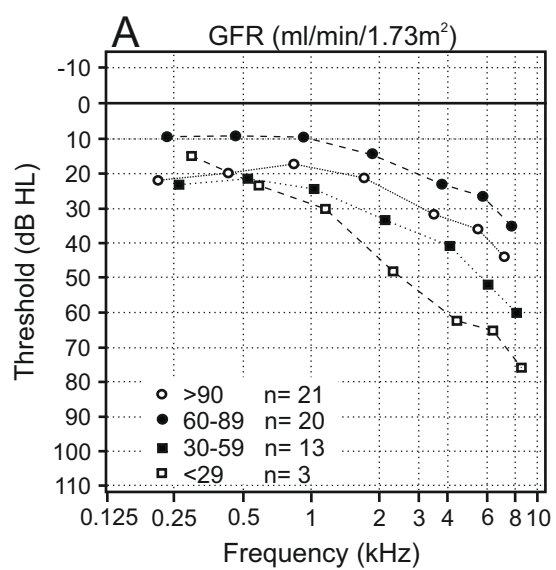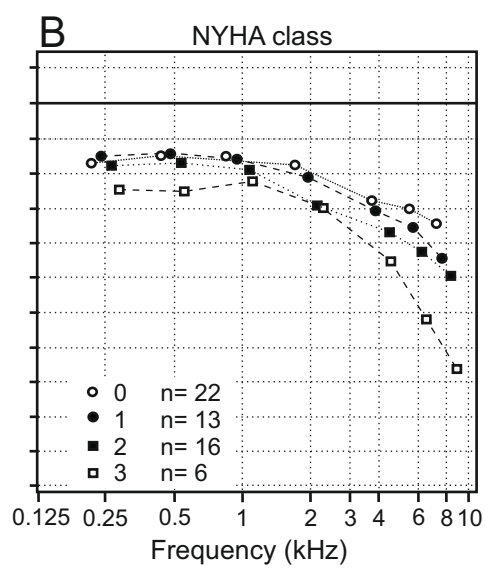

Supplement: Supplementary file 1 — Pure Tone Audiometry in relation to renal and cardiac function. Pure Tone Audiometry (AC) of the bad ear shows that hearing loss above 2 kHz is depending on the grade of GFR and NYHA. (PDF 1355 kb) [file 13023_2018_882_MOESM1_ESM.pdf]

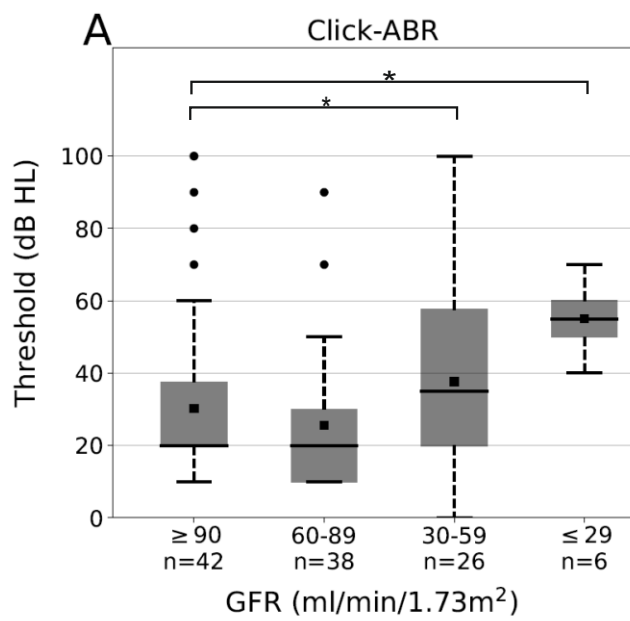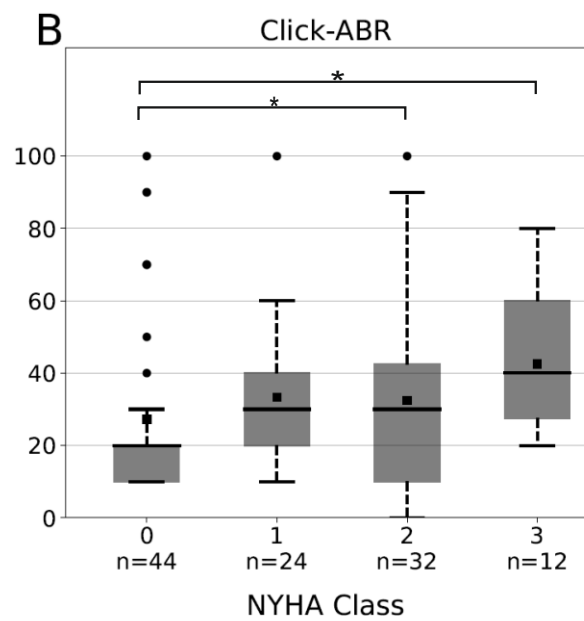

Supplement: Supplementary file 2 — ABR thresholds vs. GFR and NYHA. According (A) to progredient renal dysfunction (GFR, divided into groups: ≥90,60-89,30–59 and ≤ 29 ml/min/1.73m2) and (B) cardiac dysfunction (NYHA, divided into classes: 0,1,2,3), a statistically significant increase of ABR thresholds could be demonstrated between single groups. (PDF 1390 kb) [file 13023_2018_882_MOESM2_ESM.pdf]
